# Transitioning to adolescent and young adult or adult cancer survivorship care: A systematic review of contemporary guidelines and trials

**DOI:** 10.1007/s00520-026-10579-0

**Published:** 2026-04-08

**Authors:** Darren Haywood, Jessica Hammersley, Ursula M. Sansom-Daly, Jordana McLoone, Evan Dauer, Helen Wilding, Nicolas H. Hart, Thomas Walwyn

**Affiliations:** 1 Human Performance Research Centre, INSIGHT Research Institute, Faculty of Health, University of Technology Sydney (UTS), Driver Avenue, Moore Park, Sydney, NSW 2030 Australia; 2 Department of Mental Health, St Vincent’s Hospital Melbourne, Fitzroy, VIC Australia; 3 Department of Psychiatry, Faculty of Medicine, Dentistry and Health Sciences, University of Melbourne, Melbourne, VIC Australia; 4 School of Population Health, Faculty of Health Sciences, Curtin University, Bentley, WA Australia; 5 Centre for Mental Health and Brain Sciences, Swinburne University of Technology, Hawthorn, VIC Australia; 6 School of Nursing, College of Health and Medicine, University of Tasmania, Hobart, TAS Australia; 7 Behavioural Sciences Unit, School of Clinical Medicine, Discipline of Paediatrics and Child Health, UNSW Medicine & Health, Randwick Clinical Campus, University of New South Wales, Kensington, NSW Australia; 8 Kids Cancer Centre, Sydney Children’s Hospital, Randwick, NSW 2031 Australia; 9 Sydney Youth Cancer Service, Nelune Comprehensive Cancer Centre, Prince of Wales Hospital, Randwick, NSW 2031 Australia; 10 Library Service, St Vincent’s Hospital Melbourne, Fitzroy, VIC Australia; 11 Caring Futures Institute, College of Nursing and Health Sciences, Flinders University, Adelaide, SA Australia; 12 Exercise Medicine Research Institute, School of Medical and Health Sciences, Edith Cowan University, Perth, WA Australia; 13 Cancer and Palliative Care Outcomes Centre, Faculty of Health, Queensland University of Technology (QUT), Brisbane, QLD Australia; 14 Institute for Health Research, University of Notre Dame Australia, Perth, WA Australia; 15 Division of Paediatrics, Medical School, University of Western Australia, Perth, WA Australia; 16 Department of Paediatrics, Royal Hobart Hospital, Hobart, TAS Australia

**Keywords:** Transition, Adolescent, Young adult, AYA, Paediatric, Cancer, Survivorship, Service, Guideline, Trials

## Abstract

**Background:**

Health service transitions of child, adolescent, and young adult cancer survivors between paediatric and adult cancer care services are pervasively fragmented, resulting in poor coordination, reduced satisfaction, and adverse outcomes for cancer survivors and significant others (e.g., caregivers and family members). Numerous trials and clinical guidelines have been developed to support care transitions, but their convergence, divergence, and methodological quality remain unclear. To advance clinical practice and quality research about care transitions, a synthesis of trials and guidelines on child, adolescent, and young adult care transition across age-appropriate cancer services is required to inform future research, practice, guideline, and policy development.

**Methods:**

A systematic review (CRD420251029796) was conducted. Studies were identified from searches of six bibliographic databases, four trial registers, and grey literature. Inclusion criteria focused on clinical guidelines or trials published between January 2020 and April 2025 that addressed age-related transitional cancer care.

**Results:**

A total of 3706 records were identified. After screening, 18 records met the inclusion criteria: 10 clinical guidelines, 4 completed trials, and 4 ongoing trials with registrations. While general recommendations between guidelines were consistent, they diverged in detail relating to their local contexts. Only two ongoing trials, and no completed studies, included independent control groups.

**Conclusion:**

Completed trials were limited and highly variable in approach, components, and outcome measures, suggesting regionally specific guidelines enhance relevance, applicability, and transition practices. Future research should develop core outcome sets for care transition trials to facilitate comparison of outcomes and should develop robust study designs, including control groups.

**Supplementary Information:**

The online version contains supplementary material available at 10.1007/s00520-026-10579-0.

## Introduction

Child, adolescent, and young adult (CAYA) cancer survivors, defined as those diagnosed before 39 years of age, are at increased risk for physical and psychosocial health effects later in life (i.e., late effects), including further neoplasms, organ dysfunction, cardiometabolic disease, and lower socioeconomic status [1, 2]. To optimise their health outcomes, it is crucial to have comprehensive, well-organised long-term follow-up care programmes in place alongside adequate transition structures [3]. However, care transitions, defined as paediatric or adolescent cancer survivors transitioning to adolescent and young adult (AYA) or to adult cancer services providing survivorship care, present a complex and often challenging process, with significant implications for cancer survivors, their significant others (e.g., families, caregivers), and health professionals [4, 5]. Despite growing recognition about the importance of age-appropriate care transitions to suitable fit-for-purpose health services, a wide range of individual and system-level barriers, as well as a lack of clear transition destinations, continues to hinder optimal service navigation and continuity of care [6–8].

Child, adolescent, and young adult survivors experience barriers to engaging with adult-oriented survivorship care due to normative developmental, psychosocial, and systemic reasons [8]. Their emerging self-management and health literacy skills, potential persistent cognitive impairments, inadequate service navigation knowledge, insufficient social support, and financial constraints can hinder their capacity to understand, access, and engage with appropriate healthcare [6, 8]. These challenges are amplified by systemic issues, including inappropriate care environments, limited transition destinations, limited service capacity, and poor intra- and inter-service communication and care coordination, often resulting in CAYA survivors self-managing their own care transitions [6, 8]. Together, these barriers contribute to consistently poor experiences of care transitions when they occur, with CAYA survivors reporting low satisfaction regarding the support and services received during and after the transition process [4, 6, 7], whereby suboptimal interactions with care transition are associated with diminished quality of care and adverse health outcomes [9].

Observational and interventional research have attempted to identify and implement key considerations, actions, and processes to facilitate improved outcomes and experiences prior to, during, and after transitions of paediatric and adolescent cancer survivors to adolescent and young adult (AYA) or adult cancer services [4]. Informed by this literature and expert consensus, a multitude of clinical guidelines have been developed globally to guide effective transition into age-appropriate cancer survivorship services. However, many existing guidelines emphasise the need for additional empirical evidence, and further, the convergence and divergence across child, adolescent, young adult, and adult care transition guidelines remain largely unexamined [9]. This uncertainty extends to current and completed trials aiming to improve transition experiences and outcomes, raising important questions about how these strategies, actions and outcomes corroborate. A comprehensive synthesis of existing guidelines and trials is essential to identify best practices, highlight knowledge gaps, and inform globally relevant approaches to care transitions.

This systematic review aimed to identify, collate, describe, synthesise, and compare available clinical guidelines and trials regarding the age-appropriate transition of cancer survivors into AYA and adult cancer survivorship care. The review also sought to explore characteristics, recommendations, and evidence underpinning guidelines and trials to inform future research, practice, and guideline and policy development.

## Methods

This systematic review was registered with the International Prospective Register of Systematic Reviews (PROSPERO; ID: CRD420251029796) to ensure transparency and methodological rigour. The registration includes details of the review protocol, including objectives, eligibility criteria, data sources, and planned methods of analysis.

### Eligibility criteria

Inclusion and exclusion criteria are provided in Table 1. Clinical guidelines were defined as documents that summarise current knowledge, weigh the benefits and harms of procedures and approaches, give specific recommendations based on this information, and provide information about the evidence supporting those recommendations [10]. Trials were defined according to the National Institute of Health definition as a research study in which human subjects are assigned to interventions to evaluate the effects of those interventions on health-related outcomes [11]. To obtain the latest evidence and guidelines that are currently informing clinical practice, only documents published within the previous five years (2020 to 2025) were included.

**Table 1:** Clinical guidelines and trials eligibility criteria

Publication type	Inclusion criteria	Exclusion criteria
Clinical Guidelines	A clinical guideline, including guidance on the transition from paediatric to AYA, and paediatric or AYA to adult cancer survivorship services	Not a clinical guideline, as per the above definition Does not include guidance on the transition to AYA or adult survivorship care Full text not in the English language Conference proceedings, abstracts, and dissertations Published prior to April 2020
	Includes guidance relevant to cancer survivors who are on or off active treatment and transitioning into AYA and/or adult survivorship care	
	CAYA survivor population (i.e., diagnosed before age 39 years) to align with international variation	
Trials	Trials assessing health service transition to AYA and/or adult cancer survivorship services	Not a clinical trial Does not include a population currently transitioning, or planning to transition, to AYA or adult survivorship care Full text not in the English language Conference proceedings, abstracts, and dissertations Published prior to April 2020
	Includes cancer survivors who are on or off active treatment and transitioning into AYA and/or adult survivorship care	
	CAYA survivor population (i.e., diagnosed before age 39 years) to align with international variation	

### Information sources

Studies were identified through searches of six bibliographic databases and four trial registers: Ovid MEDLINE ALL, Embase (Ovid), Emcare (Ovid), APA PsycInfo (Ovid), CINAHL (EBSCOhost), Cochrane Library (Wiley), Clinicaltrials.gov, Australian New Zealand Clinical Trials Registry (ANZCTR), International Clinical Trials Registry Platform (ICTRP) and UK Clinical Study Registry (ISRCTN); as well as a grey literature search of guidelines, trials and registered trials through a targeted website search and Google Engine search, all ran on the 10 April 2025. Database searches were limited to 1 January 2020 onwards and performed in the English language. No limits were applied to register searches. Google Scholar notifications were also established to alert author DH to transition-focused publications following the initial data screen.

### Search strategy

#### Bibliographic databases and trial registers

Search strategies for the literature and trial registries were developed by a medical librarian (HW) in consultation with topic experts (DH and JH). A validation set of relevant publications was identified by DH and JH (see Appendix 1: Validation Set). These were text-mined using Yale MeSH Analyzer [12] and used to validate the final search strategy. Further search terms were identified through text mining in PubMed PubReminer [13]. Potential search terms retrieved were extensively tested for usefulness and relevance in Ovid Medline to develop the final search strategy, then peer reviewed by a second medical librarian and adapted for other databases. The final search strategy combined general concepts of Cancer AND Transitional Care AND (Guidelines OR Trials) using a combination of subject headings and text words. In addition, a structured search of four major clinical trial registers was conducted, using an adapted strategy of the above (see Appendix 2: Search Strategies).

Search results from bibliographic databases were exported to EndNote [14] with duplicates removed by HW. In accordance with inclusion and exclusion criteria (Table 1), records were screened on publication type by HW in EndNote. Once conference abstracts were removed, the remaining records were loaded into Covidence [15]. Each record was independently screened on title and abstract in Covidence by two independent reviewers, with six individuals contributing to the screening (DH, JH, USD, JM, ED, and TW), and with conflicts resolved by a third reviewer. Full text records were retrieved for the remaining records, which were assessed for eligibility by JH and ED, with any conflicts resolved by DH. Trials were exported to an Excel spreadsheet. Trials were screened by two independent reviewers (DH and JH) for their relevance to cancer care transition based on their title, study description, population focus, and stated objectives. Conflicts were resolved by an independent third reviewer. Only studies that explicitly addressed transitional care of CAYA cancer survivors between paediatric, AYA, and adult oncology services were considered eligible (see Table 1).

#### Google Search

As guidelines and other potentially relevant documents are commonly published on organisational and other webpages, rather than within scientific journals, a Google Search was used to identify relevant literature and resources on transition of care for CAYA cancer survivors. A structured search was conducted using search terms developed by HW, in consultation with DH and JH. Google (www.google.com) was used due to its comprehensive indexing of grey literature, government publications, and non-academic sources, and to ensure guidelines that were not published within the literature were captured. The search was conducted by JH using the search terms: cancer AND (transition OR transfer) AND (paediatric OR paediatric OR child OR adolescent OR teenage OR young OR youth) AND adult AND (guideline OR model of care OR pathway OR best practice). The search was executed on 10 April 2025, and the results were exported using a combination of manual and semi-automated techniques (see Appendix 3: Full Search Strategies). A total of 125 results were identified, exported, and screened by two independent authors (JH and DH) to assess relevance to the transition of care for CAYA cancer survivors. Screening was guided by predefined inclusion criteria (see Table 1). Each author conducted the review independently, and discrepancies were resolved through discussion.

#### Targeted Search

In line with the Google Search, within the goal of identifying relevant guidelines, a targeted search of the webpages of organisations known for publishing or endorsing evidence-based guidelines in oncology, paediatrics, and supportive care was conducted. The targeted search strategy was employed by two independent reviewers (DH and JH) who searched the target organisations' webpages on April 10, 2025, including International and Regional Health Bodies, National and Specialist Cancer Organisations, International Oncology and Supportive Care Networks and Charitable and Advocacy Organisations (see Appendix 3: Full Search Strategies). A total of 23 targeted websites were identified for screening. Each of the 23 organisational websites was independently reviewed by two authors (DH and JH) to identify clinical guidelines, models of care, frameworks, or position statements relevant to cancer care transition. Reviewers manually explored website content, including guideline repositories, publications, and policy sections, to locate freely accessible resources in line with the inclusion and exclusion criteria. Across all selection processes where consensus could not be reached, a third independent reviewer adjudicated.

### Data extraction

Data extraction was conducted independently by two authors (DH and JH), who reviewed each guideline, trial, and trial registry, and any discrepancies were assessed by a third independent reviewer. The process was guided by a set of predetermined criteria to ensure consistency and rigour (see Appendix 4: Data Extraction).

### Risk of bias and quality assessment

Included clinical guidelines were independently appraised using the Appraisal of Guidelines for Research and Evaluation II (AGREE-II) instrument [16]. Two reviewers (DH and JH) conducted assessments, evaluating six domains: Scope and Purpose, Stakeholder Involvement, Rigour of Development, Clarity of Presentation, Applicability, and Editorial Independence. All included trials were assessed using the revised Cochrane Risk-of-Bias Tool for Randomised Trials (RoB 2) [17]. Two reviewers (DH and JH) independently evaluated each study across five domains: Bias arising from the randomisation process; Bias due to deviations from intended interventions; Bias due to missing outcome data; Bias in measurement of the outcome; and Bias in selection of the reported result. Discrepancies in scoring were resolved through discussion and, where necessary, adjudicated by a third reviewer. Each guideline was provided with an overall rating on a scale of 1–7 in line with the AGREE-II tool, where the maximum (i.e., the best) possible score was 7.

## Results

### Identification and screening

**Fig. 1:**
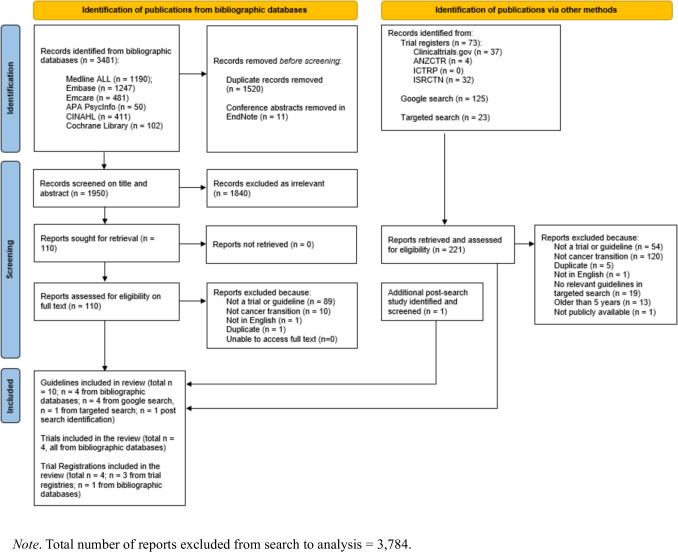
PRISMA flowchart. PRISMA, Preferred Reporting Items for Systematic Reviews and Meta-Analyses

Literature from bibliographic databases resulted in 3481 records identified. Once duplicate records and conference abstracts were removed, 1950 records had titles and abstracts screened, excluding 1840 records and retaining 110 full-text reports for full-text screening. An additional 221 records were identified through supplementary sources, including trial registries, Google search, and targeted searches, and assessed for eligibility. One large trial [18], identified through Google Scholar notifications, that satisfied the inclusion criteria was published after our search cut-off date. Given its size and relevance to the topic, and in line with best practice guidelines for systematic reviews [19–21], we included it in the analysis for this review and have noted this deviation from the original protocol. In total, 18 records met the inclusion criteria and were incorporated into the final review: 10 guidelines; 4 completed trials; and 4 trial registrations. The PRISMA flowchart is presented in Fig. 1.

### Quality appraisal

#### Clinical guidelines

Quality assessment of guidelines is presented in Table 2. Most guidelines demonstrated strong performance in the domains of Scope and Purpose, and Clarity of Presentation, reflecting clearly defined objectives and well-structured recommendations. However, variability was noted in Stakeholder Involvement and Editorial Independence, with some guidelines lacking transparency regarding funding sources or the inclusion of consumer perspectives. The Rigour of Development domain was generally well addressed, particularly in guidelines that incorporated systematic evidence reviews and formal consensus processes. The mean quality score for the included guidelines was 4.4 (SD = 0.97), indicating moderate quality.

**Table 2:** Guideline characteristics

First author	Year	Country	Age range	Guideline type	Lived experience Input	Cancer types	Settings for intended use/application	Quality^8^ (1–7)
Agency for Clinical Innovation [22]^1^	2022	Australia	< 18 years	Clinical Practice Guideline^4^	Yes	Not defined	Paediatric services; Adult services; Community-based care; Primary care; Specialist services	4
Cancer Council [23]^2^	2021	Australia	Up to 25	Optimal Care Pathway^5^	Yes	All cancer types affecting young people; rare cancers	Primary care; Tertiary cancer centres; Community and survivorship services; Palliative care	5
Cancer Council [24]	2022	Australia	15–25	Optimal Care Pathway	Yes	Acute leukaemia	Primary care; Tertiary cancer centres; Community and survivorship services; Palliative care	4
COSA [25]^3^	2025	Australia	15–25	Clinical Practice Guideline	Yes	All cancer types affecting young people	Paediatric; Adult; Survivorship clinics; Primary care; Community; Youth cancer services; Palliative care	6
Gebauer [26]	2020	Germany	<18 years	Clinical Practice Guidelines	Yes	All cancer types affecting young people; haematological malignancies	Paediatric services; Adult services; Community-based care; Primary care; Specialist services	4
Heitzer [27]	2020	United States of America	15–25	Clinical Practice Guideline	Yes	Paediatric brain tumours	Paediatric; Neuro-oncology clinics; Adult services; Survivorship clinics; Rehabilitation	4
Kerr [28]	2022	United Kingdom	16–25 years	Clinical Practice Guideline	Yes	All cancer types affecting young people	Paediatric services; Adult services; Survivorship clinics; Primary care; Community	3
Potter [29]	2024	United Kingdom	Not defined	Principles of Best Practice^6^	Yes	All cancer types affecting young people	Paediatric; Adult	4
Tonorezos [30]	2022	United States of America, Australia, England, Singapore	<19 years	Practice Recommendations^7^	Yes	All cancer types affecting young people	Paediatric; Adult; Primary care; Community; Psychosocial services	4
Wams [18]	2025	Netherlands, Switzerland, Austria, Portugal, Canada, Poland, Germany, France, Norway, England, Spain, Croatia, Ghent, Italy, Slovenia, Greece	Up to age 21 at dx	Clinical Practice Guidelines	Yes	All cancer types affecting young people	Paediatric services; Adult services; Community-based care; Primary care; Specialist services	6

^1^The Agency for Clinical Innovation is a New South Wales government organisation that leads the design and implementation of innovative models of care to improve patient outcomes and system efficiency. It works collaboratively with clinicians, consumers, and health services to drive evidence-based, patient-centred improvements across the health system

^2^The Cancer Council Australia is the nation’s leading non-government cancer control organisation. It works to reduce the impact of cancer through research funding, prevention programs, advocacy, and support services for patients and families. It also develops evidence-based guidelines and resources to improve cancer care and outcomes across Australia

^3^The Clinical Oncology Society of Australia is the peak national body representing health professionals involved in cancer care. It promotes multidisciplinary collaboration, research, education, and advocacy to improve cancer prevention, treatment, and supportive care across Australia

^4^Clinical practice guidelines are defined as systematically developed statements to assist practitioners and patients in making decisions about appropriate health care for specific clinical circumstances. They are evidence-based, transparent, and developed by multidisciplinary panels following rigorous standards

^5^Optimal care pathways are nationally endorsed framework for delivering consistent, safe, high-quality, and evidence-based cancer care across all stages of the cancer journey—from prevention and early detection through to treatment, recovery, living with chronic disease, and end-of-life care

^6^Best practice principles are evidence-informed strategies and approaches that ensure care is safe, effective, person-centred, culturally appropriate, and delivered in partnership with patients and other practitioners, using the best available evidence to achieve optimal health outcomes (Australian Commission on Safety and Quality in Health Care, 2010)

^7^Practice recommendations are statements within clinical guidelines that provide advice on what health professionals should do in specific clinical situations, based on the best available evidence, expert consensus, and consideration of benefits and harms

^8^Quality assessment scores using the AGREE-II tool, on a scale of 1–7 where 7 represents the best possible quality score

#### Trials

Risk of Bias (RoB) assessment for each trial is presented in Table 3. A mix of ‘low’ (*n* = 1) and ‘some concerns’ (*n* = 3) for risk of bias was evident across studies. Common strengths included clear reporting of outcomes and use of validated patient-reported outcome measures (PROMs) and experience measures (PREMs). Limitations included small sample sizes, lack of blinding, and incomplete reporting of allocation procedures.

**Table 3:** Trials and trials registry characteristics

First author	Year	Country	Trial type	Current status	Intervention type	Intervention approach	Lived experience	Cancer types	N	Ages	Sex/gender	Risk of bias
* Completed trials*												
Buehlmann [31]	2024	Switzerland	Single arm interventional	Complete	Model of care	Multimodal	Not reported	Leukemia/Lymphoma (41%); CNS ^1^ (18%); Other 41%	40	Age at dx: median = 8 (range 3–16); Age at baseline median = 21 (range 17–28	Sex: 29% male; 71% female	Some concerns
Carrier [32]	2025	Canada	Single arm pilot interventional	Complete	Education and skill building	Workshop	Yes	All paediatric brain tumours	12 Dyads: parents and offspring	Age at dx: mean 10.75 (range 4–14); Survivor age at assessment: mean = 17.17 (range 15–19	Gender: Survivor: male 75%; female 15%; other 8	Some concerns
Jin [33]	2023	USA^2^	Single arm interventional	Complete	Model of care	Multimodal	Yes	100% Leukaemia	33	Age at dx: median = 8 (range 1.5–32); Age at visit: median = 28 (range 19–40)	Gender: 57.6% male; 42.4% female	Some concerns
Ryan [34]	2022	Canada	Single arm interventional	Complete	Workbook	Self directed	Yes	All cancer types affecting young people	10	Aged ≥ 18 years and	Not defined	Low
* Registered trials*												
First author	Year	Country	Trial type	Current status	Intervention type	Intervention approach	Lived experience	Cancer types	*N*	Ages	Sex/gender	
Devine [35]	2025	USA	Two-arm parallel RCT^3^	Recruiting	Education	Not defined	Not defined	All cancer types affecting young people	300	Current age 18–25; dx between 0 and 19 months at least 5 years prior; at least 2 years from treatment completion	Any	
Entz-Werle [36]	2023	France	Interventional Single-Arm	Recruiting	Educational/Tool	Not defined	Not defined	All cancer types affecting young people	60	Aged ≥15 years and ≤25 years	Any	
Schmidt [37]	2022	Germany	Cluster-randomized prospective RCT	Recruiting	Model of care	Not defined	Not defined	All cancer types affecting young people	1600	Children and adolescent under 21 and one parent	Any	
Scheinemann[38]	2022	Switzerland	Interventional 3-arm non-RCT	Recruiting	Model of Care	Not defined	Not defined	All cancer types affecting young people	140	Age at dx < 18 years; Age at study ≥ 16 years	Any	

^1^Central Nervous System

^2^United States of America

^3^Randomised Control Trial

### Included document characteristics

#### Guidelines

Characteristics of included guidelines are presented in Table 2. Of the 10 clinical guidelines included, 60% (*n* = 6) were Clinical Practice Guidelines, 20% (*n* = 2) Optimal Care Pathways, 10% (*n* = 1) Principles of Best Practice, and 10% (*n* = 1) were Practice Recommendations. Regarding location, 40% (*n* = 4) were developed for Australia, 20% (*n* = 2) United Kingdom, 10% (*n* = 1) Germany, 10% (*n* = 1) United States, and 20% (*n* = 2) multinational. Target populations of the guidelines ranged from 15 to 25 years. Recommendations from guidelines (Table 4) were mapped against three transition phases (preparing for transition, during care transition, after care transition), domains of care, allowing for comparative synthesis.

**Table 4:** Guideline recommendations

Author	Preparing transition	During transition	Following transition	Domains of care	Evidence
Agency for Clinical Innovation	6 Key Principles: 1. A systematic and formal transition process including co-design with clinicians, consumers. 2. Early preparation including up-to-date medical information. 3. Empower and enable young people to self-manage. 4. Identify a local transition coordinator or facilitator. 5. Good communication and shared responsibility. 6. Individual transition plan	Maintain communication; support self-management; provide emotional and practical support; monitor progress and adjust plans as needed	Principle 7: Follow up and evaluation: Follow-up may be required for several years to ensure that each young person has engaged effectively with adult healthcare services	Physical; Psychosocial; and Cultural	Lived experience; trauma informed care; research evidence; best practice models
Cancer Council	Start early (at dx). Individualised care plans which are developmentally appropriate and are developed in conjunction with carers and family. Empowerment and education. Communicate to general practitioner treatment summary and roadmap outlining investigations and surveillance required. For patients who have undergone a transplant, transition to an adult transplantation service may be appropriate	Continuity of care; effective communication; psychosocial support; transition coordinators/navigators	Ongoing surveillance and care; long-term psychosocial wellbeing; ensure appropriate access to survivorship care plans; support for long term wellbeing and adherence to follow-up care	Physical; Psychosocial; and Cultural	Research evidence; expert consensus from haematologists, oncologists, nurses, and psychosocial professionals; Lived experience; Alignment with international best practice models, including COG^1^ and ESMO^2^ guidelines
Cancer Council	Early and proactive planning which is developmentally appropriate; individualised care plans; education and empowerment to meet the future care needs	Continuity of care; effective communication; psychosocial support; transition coordinators/navigators	5 Key principles: 1. Decisions around location of adult care for childhood cancer survivors; 2. Avoiding transition at vulnerable developmental or disease time points. 3. Avoiding transition close to stressful life events 4. A clearly identified patient navigator and written information to support transition. 5. A treatment summary and a tailored clinical surveillance roadmap	Physical (including late effects); Psychosocial; Cultural; and Spiritual	Lived experience; Research evidence; best practice models including national frameworks; expert consensus of clinicians such as oncologists, nurses
COSA	Start early (at dx). Individualised care plans which reflect a young person’s age, maturity, independence, and life stage. Multidisciplinary involvement, including psychosocial professionals, educators, family/carers, and healthcare providers. Empowerment and education	Continuity of care; support autonomy; address psychosocial needs; monitor engagement and adjust support as needed	Ongoing psychosocial care including education, employment and lifestyle Survivorship planning, evaluate outcomes and maintain connections	Psychosocial; Familial; Sexuality and reproductive; Education; Financial; Cultural and spiritual care	Research evidence; expert consensus from haematologists, oncologists, nurses, and psychosocial professionals; Lived experience; Alignment with international best practice models, including Youth Cancer Services
Gebauer	Collaborative approach between paediatric and adult care teams; psychosocial support; use of transition coordinators Early planning and a structured transition protocol. Core team developed; internal medicine specialist, psychological coworker, nurse/case manager, other specialists as required	The core team meets every 3–6 months At first late effects clinical session, risk stratification should occur	Long-term surveillance; ongoing psychosocial care and structured follow up. Every follow-up visit includes detailed consultation including but not limited to psychological screening, fertility counselling, cardiology and skeletal review	Physical; Psychosocial: Familial; Sexuality and reproductive; Education and Financial	Review of current international guidelines including COG; PanCareSurFup^3^ and European LTFU^4^ models. Data derived from German Childhood Cancer Registry
Heitzer	3 main guidelines: 1. Systematically Incorporate Neuropsychological Evaluation and Intervention. 2. Support Transitions through collaborative Multidisciplinary Input. 3. Promote the patient’s Functional Independence	Actively incorporate and utilise familial resources. Advocate for Patients in the Community	Seek patient input in transition care; to the extent they are developmentally and cognitively able to	Neurocognitive: Psychosocial; Educational and vocational: School performance, career planning, workplace accommodations Functional independence: Daily living skills, transportation, financial literacy	Best practice models (SMART^5^ Model and HCTRC^6^ Model; Research evidence including longitudinal studies on paediatric brain tumour survivors
Kerr	Early and proactive planning; individualised transition plans (PREPARATION)	Joint consults; continuity of relationships; psychosocial care; use of transition coordinators/navigators	Lifelong surveillance; ongoing psychosocial care; encourages self-management and survivor-led care planning	Physical (late effects); Psychosocial; Developmental; Educational and vocational; Reproductive health; Cultural and spiritual	Established guidelines; research evidence
Potter	Key Principles: 1. Timely identification of young people requiring transition and provision of a transition plan. 2. Provision of developmentally appropriate care that empowers young people and supports their families. 3. ID of a transition keyworker for all young people requiring transition	Key Principles: 4. Effective communication, shared responsibility, and joint working. 5. Provision of support for transition before, during and after transition to adult services. 6. A planned route for young people to access urgent advice and/or emergency care	Follow-up may be required for several years to ensure that each young person has engaged effectively with adult healthcare services	Physical; Psychosocial: Familial; and Developmental	Lived experience; trauma informed care; research evidence; best practice models
Tonorezos	Early education regarding long-term risk; structured planning Information in an easily understandable format of potential late effects and how to manage them. Communication resources provided for navigating systems and pathways	Continuity and coordination; open communication; psychosocial and fertility services. Primary care to request or elicit details of the cancer diagnosis and treatment history and addressing comorbidities	Surveillance of late effects; mental health screening; encourages self-management and survivor empowerment	Physical; Psychosocial; Cognitive; Relationships; Developmental: and Sexuality	Research evidence; international guidelines (e.g., IGHG^7^); survivor registries and cohort studies; clinical data; genomic insights into susceptibility
Wams	The transition of care process should be flexible, developmentally appropriate, and consider the medical, psychosocial, educational, and vocational needs of survivors and their families and caregivers. A transition coordinator and all other health-care providers should be involved. Written transition policy should be available at an institutional level. A transition coordinator is assigned to every survivor and their family or caregivers who develops an individual and collaborative transition plan. Institutions and international organisations provide formal, specialised, and ongoing training and education in transitional and long-term follow-up care to health-care providers who are engaged in the transition process	The actual transfer moment should: happen during a period of relative stability after evaluating the survivor’s readiness holistically. Is flexible with regard to age, on a case-by-case basis. Ensures transfer of medical records and the sharing of the transition plan. Includes a joint consultation with the survivor, current and new health-care provider (electronic sharing of information is recommended)	The quality of the transition process is evaluated at an institutional level, prioritising the needs of survivors, incorporating patient feedback, opinions, and personal experiences	Physical; Psychosocial: Familial; System navigation; and Developmental	Expert and consumer panel; research evidence; existing guideline; and peer visits

^1^Children’s Oncology Group

^2^European Society for Medical Oncology

^3^PanCare Childhood and Adolescent Cancer Survivor Care and Follow-Up Studies

^4^Long-term Follow-Up (LTFU)

^5^Specific, Measurable, Achievable, Relevant and Time-bound model

^6^Health Care Transition Research Consortium model

^7^International Guideline Harmonisation Group

#### Trials

Of the four included published trials, all were single-arm trials, with 50% (*n* = 2) conducted in Canada, 25% (*n* = 1) in Switzerland, and 25% (*n* = 1) in the United States (Table 3). The sample sizes of the published trials ranged from 10 to 40 (mean = 23.8, SD = 15.0), with a total number of 95 participants across the trials. Seventy-five percent of the published trials (*n* = 3) reported the sexes/genders of their participants, with 51.8% (*n* = 44) of participants across these trials being female. Most of the published trials either reported the mean (25%, *n* = 1) or median (50%, *n* = 2) age of participants at the time of diagnosis and assessment. Median and mean age at diagnosis was 8 years and 10.75 years, respectively; whereby the range of ages of participants at diagnosis was 1.5 years to 32 years. Seventy-five percent (75%, *n* = 3) of the published trials reported the cancer types of their participants, with 25% (*n* = 1) of included trials focusing exclusively on brain tumours, 25% (*n* = 1), 25% on focusing exclusively on Leukemia, and 25% (*n* = 1) including multiple cancer types (primarily Leukemia/Lymphoma = 41% and central nervous system = 18%). Of the included trials, 75% (*n* = 3) reported lived-experience intervention in their design and delivery. Half of the trials (50%; *n* = 2) utilised a multimodal intervention (multi-pronged model of care), 25% (*n* = 1) workshop intervention (education and skill building), and 25% (*n* = 1) self-directed planning intervention (workbook). The primary outcome domains of assessment varied considerably across the trials. Across the four trials, primary outcome domains included transition readiness (25%, *n* = 1), perceived self-management ability (25%, *n* = 1), cancer worries (25%, *n* = 1), confidence and self-efficacy (50%, *n* = 2), knowledge (25%, *n* = 1), as well as indices related to evaluations of the transition intervention itself, including understandability and actionability (25%, *n* = 1), and overall impression of the intervention (25%, *n* = 1).

The four included registered current trials are presented in Table 3, of which 50% (*n* = 2) were randomised control trials, 25% (*n* = 1) were single-arm trials, and 25% (*n* = 1) were quasi-experimental (non-randomised) 3-arm trials. Twenty-five percent (*n* = 1) are being conducted in the United States of America, 25% (*n* = 1) in Germany, 25% (*n* = 1) in France, and 25% (*n* = 1) in Switzerland. The target sample sizes of the registered current trials ranged from 60 to 1600 (mean = 525.0, SD = 732.6), with a total number of 2100 target participants across the trials. None (0%, *n* = 0) of the registered current trials specified particular target sexes/genders, and 50% (*n* = 2) specified a target age range at diagnosis (0–19 months of age, and =  <18 years, respectively). All trials specified their target age, which differed across each individual trial but ranged between 15 and 25 years (see Table 3). None of the registered current trials specified specific cancer types targeted nor lived-experience input in the development or conduct of the trial. Fifty percent (*n* = 2) of registered current trials are utilising a multimodal intervention (multi-pronged model of care), and 50% (*n* = 2) are utilising an educational intervention (knowledge and skill building). Like the published trials, the primary outcome domains of assessment varied considerably across the registered current trials, with 10 different outcomes being measured (see Table 3). The only endpoint being measured by more than one trial is self-management skills, being measured across two trials currently underway (50%). Aims and outcomes of the completed trials and the aims and efficacy outcomes to be assessed for the current trials are presented in Tables 5 and 6, respectively.

**Table 5:** Trial aims and outcomes

First Author	Aim	Efficacy	Acceptability	Key Findings
Buehlmann	Examine the evolving needs and level of cancer-related knowledge among CCS^1^ during their transition from paediatric to adult follow-up care, using longitudinal data from the ACCS^2^ project	Cancer knowledge: most CCSs stated to recall their type of cancer (88%), the cancer location (94%), and their age at diagnosis (100%) at baseline. The proportion of CCSs being sure about how often follow up visits take place decreased from baseline (88%) to 15 months (76%). The proportion of CCSs who stated to know some, or all potential late effects increased from 35 to 82%. Potential risk for late effects:, 35–53% of CCSs reported being unsure at baseline. This proportion decreased for all organ systems except for secondary malignancies, where it remained at 53%. The largest increase in knowledge could be shown for late effects of the heart, ear, eyes and bones with an approximation of the physicians’ values. The physicians rated the risk for secondary malignancies (94%), fertility (88%), and bone health (71%) as endangered most frequently. Cancer Worries: CCSs showed moderate worries with a median CWS^3^ score of 62, which was identical at baseline and after transition. e leukemia survivors had an incline in the median CWS-Score of 17 (56.5 at baseline, 73.5 at 15 months), the survivors of other cancers had a decline of 4 (62 at baseline, 58 at 15 months). Self-Management: At baseline, CSSs (strongly) agreed to 5 of the 15 SMSS^4^ statements with at least 90%, and with 75% to 90% to 6 additional statements. The results were similar after transition	85% of participants rated transition interventions (education sessions, written materials) as helpful or very helpful. 78% preferred digital tools for ongoing education. 90% supported the idea of structured transition programs integrated into routine care	Knowledge Gaps: Only 58% of survivors could correctly identify their cancer diagnosis;46% knew their treatment history (e.g., chemotherapy type); Less than 40% were aware of potential late effects such as fertility issues or cardiac risks. Transition Needs: 72% reported needing more information about long-term health risks; 65% wanted guidance on navigating adult healthcare systems; Psychosocial support needs increased significantly during transition (*p* < 0.05). Education Impact: Survivors who received structured education during paediatric care scored 25% higher on knowledge assessments compared to those without formal education
Carrier	Evaluate whether structured, targeted workshops can improve transition readiness among paediatric brain tumour survivors as they move from paediatric to adult care	Not statistically significant improvement in PBTSTRAQ^5^ (transition readiness) scores was observed from the point of view of PBTS (*t*(9) = −1.14, *p* = 0.14, *d* = 0.36) as well as their parents (*t*(8) = −0.75, *p* = 0.24, *d* = 0.25). A statistically significant improvement following the workshops was only observed for PBTS self-efficacy (*t*(9) = −2.079, *p* = 0.03, *d* = 0.66), with a moderate effect size	Most PBTS^6^ participants (73%) rated the workshop program as “acceptable” or “very acceptable” PBTS expressed the most satisfaction was on social skills and peer relations (2nd workshop; 79%). The PBTS participants expressed the least satisfaction was the one pertaining to cognitive challenges and return to daily activities (3rd workshop; 61%). Overall satisfaction among parents, an average of 86% of parents rated the workshop program as “acceptable” or “very acceptable”. Parent participants expressed the most satisfaction were those on social skills and peer relations (2nd workshop; 88%) and on cognitive challenges (3rd workshop; 88%). Parent participants expressed the least satisfaction with the workshop on disease self-management (1st workshop; 83%). Notably, on average, 97% of parents and 98% of young people responded that they described the workshop programs “neutral”, “acceptable”, or “very acceptable”	Measured using Transition Readiness Assessment Questionnaire Clinically relevant improvements in transition readiness skills. PBTS: effect size *d* = 0.36. Caregivers: effect size *d* = 0.25. Changes were not statistically significant due to small sample size but promising for future research
Jin	Develop, implement, and evaluate a structured pilot clinic designed to support childhood and adolescent cancer survivors as they transition from paediatric oncology care to adult survivorship care	Structured Interview: Overall impression: positive 95%, mixed 5%. Paediatric-to-adult transition quality before GREAT^7^ Survivorship Clinic visit: Adequate 25%, inadequate/suboptimal 75%. Changes in confidence managing health after GREAT Survivorship Clinic visit: improved 81%. Not changed 19%, worsened 0%. Recommend clinic to other cancer survivors 91%, Undecided 9%, No 0%	Patients: Expressed strong satisfaction with the clinic model, noting improved confidence in managing their health and navigating adult care. PCPs: Valued the structured communication and coordination provided by the clinic, which helped clarify survivorship care plans. The clinic was considered feasible, practical, and beneficial, supporting its potential for broader implementation	Clinic improved: Self-management skills; Care coordination between oncology and primary care. Highlighted need for innovative strategies to connect survivors with primary care providers
Ryan	Assess the feasibility, acceptability, and preliminary effectiveness of a structured transition workbook designed to support childhood cancer survivors as they move from paediatric to adult care	Understandability and actionability PEMAT-P^8^ tool: The overall PEMAT-P score and the standard deviation were 94.06 ± 7.40. The mean PEMAT-P score and standard deviation (SD) for understandability were 92.83 ± 8.79and actionability was 98.15 ± 5.24	Understandable: 92.83% (SD ± 8.79). Actionable: 98.15% (SD ± 5.24). Overall PEMAT-P score: 94.06% (SD ± 7.40)	Overall PEMAT-P score: 94.06 (SD ± 7.40). Understandability: 92.83 (SD ± 8.79). Actionability: 98.15 (SD ± 5.24). Strong interrater reliability Participants reported high satisfaction and supported transition-focused interventions

^1^Childhood Cancer Survivors

^2^Aftercare of Childhood Survivors in Switzerland Project

^3^Cancer Worry Score

^4^Self-management skills scale

^5^Paediatric brain tumour survivors Transition Readiness Assessment Questionnaire

^6^Paediatric brain tumour survivors

^7^Getting Regular Evaluations After Treatment

^8^Patient Education Materials Assessment Tool for Printable Material

**Table 6:** Trials registries aims and outcome measurements

First author	Aim	Efficacy
Devine	To evaluate the efficacy of a digital self-management and peer mentoring intervention in improving survivorship care engagement among young adult survivors of childhood cancer	Health-related Quality of Life: baseline 3 months, 12 months. The Patient Reported Outcomes Measurement Information System (PROMIS) Global Health. Self-Management behaviours: Baseline, 3 months, 12 months. The Self-Management Skills scale
Schmidt	To evaluate the effectiveness of a structured, multidisciplinary, guideline-based long-term aftercare program in improving self-efficacy and health outcomes among childhood and adolescent cancer survivors, compared to standard care	Self-efficacy of parents of affected children and adolescents after intervention: General self-efficacy expectancy scale (baseline, after intervention, 3 months after intervention). Adolescents’ transition readiness from age 14 incl. autonomy; health literacy; adherence; Active participation of patients in the treatment process; satisfaction with care among parents and children; health-related quality of life; behavioural problems; and health status
Entz-Werle	To assess the feasibility and user experience of a mobile health application (OnKO-TnT) designed to support adolescents and young adults with cancer in their therapeutic and educational journey	Global usability score from the mHealth App Usability questionnaire (21 items), completed by participants after 6 months of using the app
Scheinemann	To evaluate the current needs, knowledge, and transition experiences of adolescent and young adult childhood cancer survivors, and to compare different models of long-term follow-up care to improve adherence and health outcomes	Cancer Worry: Cancer Worry Scale (baseline 3 months, 15 months). Self-management: Self-Management Skill Scale (baseline, 3 months, 15 months). Evaluation of needs for ongoing care: “Scale for ongoing Care” (baseline, 3 months, 15 months). Evaluation of expectation for transition: Expectation Scale (baseline, 3 months, 15 months). Survivors' cancer specific knowledge (baseline, 3 months, 15 months)

### Narrative synthesis

**Fig. 2:**
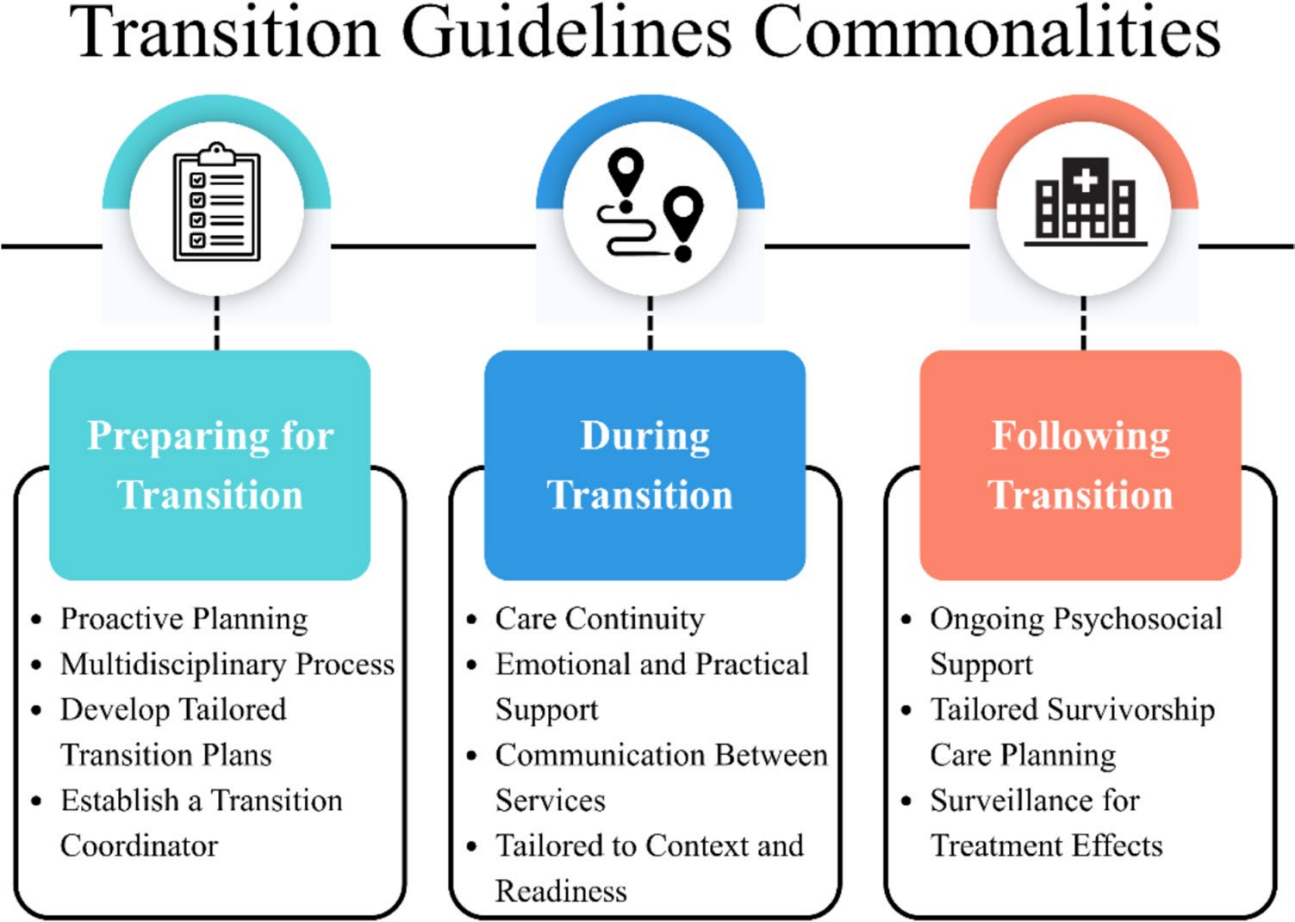
Key overarching commonalities across the guidelines. *Note.* This figure presents key overarching commonalities among a number of the guidelines. It is not exhaustive and does not represent the content of each guideline.

Figure 2 presents key overarching commonalities across the guidelines, which are explored in greater depth in the sections below, alongside a discussion of notable differences.

#### Guidelines

##### Preparing for transition

Preparation for health service or care transition is universally recognised as a foundational phase in ensuring continuity and quality of care for young people moving from paediatric to adult health services [39, 40]. Across guidelines, early and proactive planning is emphasised as essential to mitigating risks and promoting engagement (see Table 4). Kerr et al. [28], Potter et al. [29] and the Clinical Oncology Society of Australia (COSA) [25] advocate for the development of individualised transition plans that are responsive to the young person’s developmental stage, psychosocial context, and future care needs. These plans are ideally initiated at diagnosis and refined over time. The Agency for Clinical Innovation [22] proposes a structured framework focusing on early preparation. Cancer Council [23, 24] and Tonorezos et al. [30] similarly stress the importance of education regarding long-term risks and the provision of communication tools to support navigation of adult services. Gebauer et al. [26] and Wams et al. [18] extend this approach by recommending the formation of multidisciplinary care teams and institutional policies to support health service and care transition planning. The consistent recommendation to appoint a care transition coordinator [18, 26, 29] reflects a growing consensus on the need for dedicated roles to facilitate continuity and advocacy. Several guidelines draw on lived experience and trauma-informed care principles [22, 29], while others are informed through multidisciplinary expert consensus and international frameworks.

##### During care transition

Health service and care transition phases are characterised by a need to maintain continuity, foster autonomy, and ensure psychosocial safety. COSA [25], Cancer Council [23, 24] and Heitzer et al. [27] highlight the importance of sustained relationships and the provision of emotional and practical support. Effective communication between paediatric, AYA, and adult cancer services is a central theme in the guidelines by Potter et al. [29] and Tonorezos et al. [30], who also advocate for shared responsibility and joint working models. Kerr et al. [28] and Wams et al. [18] recommend joint consultations and flexible timing of transfer, tailored to individual readiness and stability; and Gebauer et al. [26] propose a structured model involving regular multidisciplinary meetings and risk stratification at the initial late effects consultation. Heitzer et al. [27] emphasise the integration of familial resources and community advocacy, particularly for survivors of paediatric brain tumours. These approaches reflect a shift toward holistic, person-centred care that is well supported by expert consensus.

##### Following care transition

Post-transition management is increasingly recognised as a long-term process requiring sustained engagement, surveillance, and evaluation. Kerr et al. [28], Tonorezos et al. [30], and Gebauer et al. [26] recommended ongoing psychosocial support, survivorship planning, and structured follow-up that includes ongoing surveillance for treatment-related complications, including cardiac toxicity, pulmonary dysfunction, endocrine disorders, neurocognitive impairment, infertility, and secondary malignancies, as well as the psychological wellbeing. Cancer Council [23, 24] and COSA [25] advocated for access to tailored survivorship care plans and support mechanisms to promote adherence and wellbeing. Heitzer et al. [27] extend this model by incorporating neuropsychological evaluation and vocational planning, with a focus on promoting functional independence. Wams et al. [18] and the Agency for Clinical Innovation [22] emphasise institutional-level evaluation of transition quality, incorporating survivor feedback and outcome data to inform continuous improvement.

##### Domains of care

Guidelines span a wide array of care domains, underscoring the complexity and multidimensionality of care and health service transition planning, including but not limited to physical health and late effects, psychosocial and emotional wellbeing, familial and cultural considerations, educational, vocational, and financial support, and spiritual and developmental needs.

### Trials and trial registries

#### Intervention design and delivery

Despite methodological variation, all interventions aimed to enhance autonomy, self-management, and long-term engagement. Digital and hybrid models featured prominently in both current and published trials (60%). For example, Devine et al. [35] are piloting a mobile-based self-management and peer mentoring programme for young adults, with an equity lens through Hispanic oversampling. Further, Entz-Werle et al. [36] are evaluating *OnKO-TnT*, a mobile app supporting AYA survivors’ therapeutic and educational navigation. Ryan et al. [34] developed a structured workbook (*Life After the Janeway*), while Carrier et al. [32] delivered psychoeducational workshops for paediatric brain tumour survivors (PBTS) and caregivers.

Structured care pathways were also commonly engaged. Jin et al. [33] implemented the *GREAT Survivorship Clinic*, combining medical review with AYA-specific counselling and primary care provider (PCP) liaison. Schmidt et al. [37] are trialling a multidisciplinary, guideline-concordant model with psychosocial counselling, while Scheinemann et al. [38] are assessing, and Buehlmann et al. [31] have assessed, longitudinal transition pathways across Swiss cancer centres. All interventions addressed multiple domains: medical, psychosocial, cognitive, vocational, and reproductive.

#### Key outcomes

Across studies, patient-reported outcomes were especially prevalent. Improvements were noted in transition readiness [32], self-efficacy [32], and cancer knowledge [31]. Jin et al. [33] reported enhanced confidence and PCP communication, while Ryan et al.’s workbook scored highly on usability (PEMAT-P: 94%) [34]. However, persistent gaps were identified with awareness of fertility and secondary malignancy risks remaining low [31], and systemic barriers such as insurance navigation and PCP linkage persist [33]. Regarding current trials, similar key outcome domains as the published trials are typically being used. For example, Entz-Werle et al. [36] are examining engagement and perceived benefit, and Schmidt et al. [37] are assessing self-efficacy.

#### Feasibility and acceptability

All studies that assessed health service and care transition intervention feasibility and acceptability demonstrated positive results. For example, retention was strong (e.g., 83% in Carrier et al. [32]), and satisfaction ratings were consistently high (e.g., 95% in Jin et al. [33]). Digital tools were well received but required attention to technical quality and interactivity. Recruitment challenges were common, particularly among underserved populations, highlighting the importance of equity-focused strategies.

## Discussion

This review provided a synthesis of clinical guidelines and completed or ongoing trials about age-appropriate health service and care transition for CAYA cancer survivors to adult cancer services to inform future research, practice, guideline, and policy development. The review is particularly timely, as there is increasing recognition of the critical importance of implementing evidence-based, high-quality transition practices to improve both the experiences and outcomes of young people living with and beyond cancer [29, 41]. This review shows that there are multiple guidelines published across different global regions, which include guidance on supporting age-appropriate transition for young people affected by cancer. Guidelines focused specifically on health service or care transition such as providing patients with joint consults and collaborative approaches between paediatric and adult care teams [26, 28, 30], as well as broader guidelines which include care guidance during the health service transition process such as the use of shared-decision making and structured support to promote long-term wellbeing and adherence to follow-up care [25]. Relatedly, there are guidelines that are aimed to be applicable internationally [18], as well as those that are focused on a specific country [23, 24]. 

Existing clinical guidelines were largely consistent with regard to their recommendation domains, and aligned their recommendations to address individual-level barriers to high-quality transition identified in the literature [6–8, 41], including cancer survivor self-management skills, service navigation knowledge, social support, as well as system-level barriers, including adult care environment, capacity, information sharing, communication, and coordination between service types. In line with addressing qualitative and quantitative evidence associated with poor transition experiences [6–8, 41], a key recommendation between the guidelines is to engage with all stakeholders, including the cancer survivor, parents/caregivers, and the multidisciplinary team at current and destination services, early to proactively plan transition. Relatedly, the guidelines consistently recommended that the cancer survivor and parents or caregivers be active participants in the transition planning process with the multidisciplinary team. This recommendation seeks to mitigate occurrences of poorly tailored transition planning, communication, and health service interactions, which are associated with negative transition experiences as well as poorer quality of life and health outcomes [4, 6, 7, 42]. However, the guidelines differ in terms of the detail provided within their recommendations and how explicit the recommendations are. This is largely driven by the breadth of the guideline, including whether it is an international guideline or targeted to a particular geographic region, resulting in differences in health systems and resources. Given concerns about the practicality and uptake of clinical guidelines in routine practice across oncology [43, 44], international guidelines may offer useful general recommendations, while more localised guidelines are required to guide specific actions and processes in practice.

Early and structured preparation of the young person ahead of their healthcare transition was consistently identified as a target in the interventions, with repeated assessments of readiness and autonomy-building activities embedded throughout the transition process in multiple completed and current trials [31–34]; however, services specifically available for those transitioning into adult services were not clearly outlined. In line with the guidelines and empirical literature [4, 5, 41] championing multidisciplinary holistic transitional care, the majority of the included trials included components that addressed multiple domains of wellbeing, including medical, psychosocial, cognitive, vocational, and reproductive. The range and diversity of care domains covered in this review reflect a paradigm shift from disease-centred to person-centred care, recognising that successful transition requires attention to the full spectrum of health, identity, and life course development. We also observed some possible trends in terms of intervention modality and approach. In recent years, digital tools and approaches have gained strong interest within cancer survivorship care due to improved accessibility and potential efficacy [45–47]. Most trials included here utilised either digital or hybrid delivery models. However, in line with the body of literature in oncology utilising digital approaches [45–47], while satisfaction and acceptability were found to generally be strong, there were mixed findings with regard to usability, quality, and interactivity of the digital tools. While the trials largely aligned with general recommendations provided in the guidelines, the characteristics and components of the interventions were highly variable, demonstrating the breadth of the typical recommendations provided in the guidelines.

Our findings must be considered in the context of several limitations of the current literature. The completed trials were single-arm interventions; therefore, they did not include an independent control group and included a variety of different outcomes and measurements. Future research should develop core outcome sets for health service or care transition trials to facilitate comparison. These could build on recently developed person-centred core outcome sets to measure the quality of adolescent and young adult cancer care more broadly [48]. Given this, along with the variable intervention characteristics and components, it is challenging to draw conclusions on the efficacy and utility of specific interventions and approaches. Furthermore, none of the completed or current trials examined transition from paediatric services to a care destination of an adolescent and young adult-specific service, perhaps due to the dichotomy of paediatric and adult hospitals and health services worldwide. There are considerable developmental and psychosocial differences between AYA-aged survivors of a cancer diagnosed in childhood, as opposed to adult survivors of a cancer diagnosed during the AYA years; yet currently, the evidence base around transition is not mature enough to enable examinations of intervention impact according to these specific developmental stages [49]. It will also be important for future research to closely attend to developmentally appropriate transition models along the continuum of child and AYA cancer survivors. Of the ongoing trials that are currently being conducted, three out of four include an independent comparison group, and two out of three are RCTs across paediatric, adolescent, and young adult cancer patient groups. This suggests that future research in this area may be able to facilitate stronger conclusions.

### Future directions to improve age-appropriate cancer care transition

To further strengthen age-appropriate cancer care transition, this review has highlighted the need to map existing transition approaches at a service provision level, evaluating their scope, quality, and clarity regarding the destination of care for CAYA cancer survivors. This mapping exercise, combined with insights from the current systematic review and extensive consumer and user involvement, should inform the development of future nationally endorsed guidelines to standardise and enhance cancer transition practices, providing actionable, evidence-based recommendations to reduce variability in care and improve long-term outcomes and follow-up for patients.

Developmental trajectories among CAYA survivors are variable, and some individuals do not achieve typical milestones of independence due to the enduring effects of cancer and its treatment. Transition models should therefore adopt a needs-led, developmentally informed approach that prioritises functional and psychosocial needs. Many adult services lack structured survivorship programmes, leaving survivors to manage complex late effects and psychosocial needs without specialised support. This gap highlights the need for advocacy, policy reform and innovative models of care to ensure continuity and equity in survivorship. In parallel, future research should prioritise rigorous randomised control trials to evaluate structured transition interventions, ensuring guideline development is underpinned by high-quality empirical evidence and adaptable to diverse health system contexts. Collectively, these efforts will bridge the gap between transition readiness and actual access to survivorship care, creating a sustainable, patient-centred framework for CAYA cancer survivors.

## Conclusion

This systematic review synthesised the highest level of contemporary evidence in the domain of age-appropriate transitional care for young people affected by cancer. Several guidelines exist to support transitional care, which are largely aligned in their recommendations, but will benefit from local tailoring to enhance their translation into practice. Only a small number of trials have been completed to improve transition practice, which were mostly highly variable in their approach, components, and outcome measures, included small samples, and lacked control groups, making it challenging to draw conclusions. Future rigorous work is needed to inform optimal transition to survivorship care for child and AYA cancer survivors.

## Supplementary Information

Below is the link to the electronic supplementary material.

Supplementary material 1 (PDF 229 KB)

## Data Availability

Not applicable.
