# Supplementary material for: Transitioning to adolescent and young adult or adult cancer survivorship care: A systematic review of contemporary guidelines and trials
Source: Support Care Cancer. 2026 Apr 8;34(5):411. doi: 10.1007/s00520-026-10579-0 (PMC13061783; doi:10.1007/s00520-026-10579-0)
Supplement: Supplementary file 1 — Supplementary material 1 (PDF 229 KB) [file 520_2026_10579_MOESM1_ESM.pdf]

TRANSITIONING TO ADOLESCENT AND YOUNG ADULT OR ADULT CANCER SURVIVORSHIP CARE: A SYSTEMATIC REVIEW OF CONTEMPORARY GUIDELINES AND TRIALS

Appendices

Contents

Appendix 1: Validation set of papers....................2
Appendix 2: Search Strategies....................3
Appendix 3: Full search strategies....................9
Appendix 4: Data extraction....................11

Appendix 1: Validation Set of Papers

Effinger, K. E., Haardorfer, R., Marchak, J. G., Escoffery, C., Landier, W., Kommajosula, A., . . . Mertens, A. C. (2023). Current pediatric cancer survivorship practices: a report from the Children's Oncology Group. J Cancer Surviv, 17(4), 1139-1148. doi:10.1007/s11764-021-01157-w

Ehrhardt, M. J., Friedman, D. N., & Hudson, M. M. (2024). Health Care Transitions Among Adolescents and Young Adults With Cancer. J Clin Oncol, 42(6), 743-754. doi:10.1200/JCO.23.01504

Ekaterina, A., Thorsten, L., Gabriele, C., Juliane, G., Swart, E., & Baust, K. (2025). Stepping into adulthood: pediatric cancer survivors and their parents' perspectives on the transition from pediatric to adult care. BMC Health Serv Res, 25(1), 204. doi:10.1186/s12913-025-12326-3

Freyer, D. R. (2010). Transition of care for young adult survivors of childhood and adolescent cancer: rationale and approaches. J Clin Oncol, 28(32), 4810-4818. doi:10.1200/JCO.2009.23.4278

Marchak, J. G., Sadak, K. T., Effinger, K. E., Haardorfer, R., Escoffery, C., Kinahan, K. E., . . . Mertens, A. (2023). Transition practices for survivors of childhood cancer: a report from the Children's Oncology Group. J Cancer Surviv, 17(2), 342-350. doi:10.1007/s11764-023-01351-y

Mulder, R. L., van der Pal, H. J. H., Levitt, G. A., Skinner, R., Kremer, L. C. M., Brown, M. C., . . . Frey, E. (2016). Transition guidelines: An important step in the future care for childhood cancer survivors. A comprehensive definition as groundwork. Eur J Cancer, 54, 64-68. doi:10.1016/j.ejca.2015.10.007

Osborn, M., Johnson, R., Thompson, K., Anazodo, A., Albritton, K., Ferrari, A., & Stark, D. (2019). Models of care for adolescent and young adult cancer programs. Pediatr Blood Cancer, 66(12), e27991. doi:10.1002/pbc.27991

Otth, M., Denzler, S., Koenig, C., Koehler, H., & Scheinemann, K. (2021). Transition from pediatric to adult follow-up care in childhood cancer survivors-a systematic review. J Cancer Surviv, 15(1), 151-162. doi:10.1007/s11764-020-00920-9

Schwartz, C. L. (2020). Creating a bridge for transition: From pediatric cancer survival to life-long, risk-based health care of the adult cancer survivor. Cancer, 126(3), 473-476. doi:10.1002/cncr.32569

Viner, R. (2003). Bridging the gaps: transition for young people with cancer. European Journal of Cancer, 39(18), 2684-2687. doi:10.1016/j.ejca.2003.08.004

Appendix 2: Search Strategies

Ovid MEDLINE(R) ALL 1946 to April 08, 2025

1. exp Neoplasms/ or exp Medical Oncology/ or Oncology Service, Hospital/ or Oncology Nursing/ or Cancer Survivors/
2. (cancer* or carcinoma* or adenocarcinoma* or blastom* or glioblastom* or glioma* or leukaem* or leukem* or lymphom* or malignan* or melanoma* or metastas* or mesothelio* or mesotelio* or myeloma* or neoplas* or neuroblastom* or oncolog* or osteo?sarcom* or sarcom* or tumo?r* or glioblastoma*).ti,ab,kf.
3. 1 or 2
   ransition to adult care/
4. (transition* and (adult* or adolescen* or child* or p?ediat* or teenag* or young or youth*)).ti.
5. ((transition* or transfer*) adj2 (adult* or adolescen* or child* or p?ediat* or teenag* or young or youth*)).ti,ab,kf.
6. (adolescen* or child* or p?ediat* or teenag* or young or youth*).ti.
7. (adolescent/ or child/ or young adult/) and adult/
8. 7 or 8
9. "continuity of patient care"/ or aftercare/ or patient handoff/ or patient transfer/ or transitional care/ or (aftercare or after care or continuity of care or follow up or transition*).ti,kf.
10. 9 and 10
11. 4 or 5 or 6 or 11
12. guideline/ or practice guideline/ or Guideline Adherence/ or Practice Guidelines as Topic/ or "Standard of Care"/ or "Delivery of Health Care"/ or models, organizational/
13. (guideline* or clinical protocol* or clinical standard* or consensus statement* or framework* or model* or pathway* or policies or policy or practice* or "standard* of care" or statement*).ti,ab,kf.
14. clinical study/ or exp clinical trial/ or clinical trial protocol/ or observational study/ or comparative study/ or evaluation study/ or multicenter study/
15. epidemiologic studies/ or exp case-control studies/ or exp cohort studies/
16. (trial* or study or rct).ti,ab,kf.
17. 13 or 14 or 15 or 16 or 17
18. 3 and 12 and 18
19. exp animals/ not humans/
20. news.pt.
21. 20 or 21
22. 19 not 22
23. limit 23 to (english language and yr="2020 -Current")

Embase 1974 to 2025 April 08 (Ovid)

1. exp malignant neoplasm/ or exp oncology/ or exp oncology nursing/ or cancer center/ or cancer patient/ or cancer survivor/ or childhood cancer survivor/

2. (cancer* or carcinoma* or adenocarcinoma* or blastom* or glioblastom* or glioma* or leukaem* or leukem* or lymphom* or malignan* or melanoma* or metastas* or mesothelio* or mesotelio* or myeloma* or neoplas* or neuroblastom* or oncolog* or osteo?sarcom* or sarcom* or tumor* or tumour* or glioblastoma*).ti,ab,kf.
3. 1 or 2
4. transition to adult care/
5. (transition* and (adult* or adolescen* or child* or p?ediat* or teenag* or young or youth*)).ti.
6. (transition* or transfer*) adj2 (adult* or adolescen* or child* or p?ediat* or teenag* or young or youth*)).ti,ab,kf.
7. (adolescen* or child* or p?ediat* or teenag* or young or youth*).ti.
8. (juvenile/ or adolescent/ or child/ or young adult/) and adult/
9. 7 or 8
10. transitional care/ or aftercare/ or clinical handover/ or (aftercare or after care or continuity of care or follow up or transition*).ti,kf.
11. 9 and 10
12. 4 or 5 or 6 or 11
13. exp practice guideline/
14. (guideline* or clinical protocol* or clinical standard* or consensus statement* or framework* or model* or pathway* or policies or policy or practice* or "standard* of care" or statement*).ti,ab,kf.
15. clinical study/ or exp clinical trial/ or intervention study/ or exp longitudinal study/ or major clinical study/ or prospective study/ or retrospective study/
16. (trial* or study or rct).ti,ab,kf.
17. 13 or 14 or 15 or 16
18. 3 and 12 and 17
19. exp animal/ not human/
20. (book or chapter or conference abstract or conference paper or "conference review" or note).pt.
21. 19 or 20
22. 18 not 21
23. limit 22 to (english language and yr="2020 -Current")

Ovid Emcare 1995 to 2025 Week 14

1. exp malignant neoplasm/ or exp oncology/ or exp oncology nursing/ or cancer center/ or cancer patient/ or cancer survivor/ or childhood cancer survivor/
2. (cancer* or carcinoma* or adenocarcinoma* or blastom* or glioblastom* or glioma* or leukaem* or leukem* or lymphom* or malignan* or melanoma* or metastas* or mesothelio* or mesotelio* or myeloma* or neoplas* or neuroblastom* or oncolog* or osteo?sarcom* or sarcom* or tumor* or tumour* or glioblastoma*).ti,ab,kf.
3. 1 or 2
4. transition to adult care/
5. (transition* and (adult* or adolescen* or child* or p?ediat* or teenag* or young or youth*)).ti.

6. (transition* or transfer*) adj2 (adult* or adolescen* or child* or p?ediat* or teenag* or young or youth*)).ti,ab,kf.
7. (adolescen* or child* or p?ediat* or teenag* or young or youth*).ti.
8. (juvenile/ or adolescent/ or child/ or young adult/) and adult/
9. 7 or 8
10. transitional care/ or aftercare/ or clinical handover/ or (aftercare or after care or continuity of care or follow up or transition*).ti,kf.
11. 9 and 10
12. 4 or 5 or 6 or 11
13. exp practice guideline/
14. (guideline* or clinical protocol* or clinical standard* or consensus statement* or framework* or model* or pathway* or policies or policy or practice* or "standard* of care" or statement*).ti,ab,kf.
15. clinical study/ or exp clinical trial/ or intervention study/ or exp longitudinal study/ or major clinical study/ or prospective study/ or retrospective study/
16. (trial* or study or rct).ti,ab,kf.
17. 13 or 14 or 15 or 16
18. 3 and 12 and 17
19. exp animal/ not human/
20. (book or chapter or conference abstract or conference paper or "conference review" or note).pt.
21. 19 or 20
22. 18 not 21
23. limit 22 to (english language and yr="2020 -Current")

APA PsycInfo 1806 to March 2025 Week 5

1. exp neoplasms/ or exp oncology/
2. (cancer* or carcinoma* or adenocarcinoma* or blastom* or glioblastom* or glioma* or leukaem* or leukem* or lymphom* or malignan* or melanoma* or metastas* or mesothelio* or mesotelio* or myeloma* or neoplas* or neuroblastom* or oncolog* or osteo?sarcom* or sarcom* or tumor* or tumour* or glioblastoma*).ti,ab.
3. 1 or 2
4. (transition* and (adult* or adolescen* or child* or p?ediat* or teenag* or young or youth*)).ti.
5. ((transition* or transfer*) adj2 (adult* or adolescen* or child* or p?ediat* or teenag* or young or youth*)).ti,ab.
6. early adolescence/ or emerging adulthood/ or late adolescence/ or (adolescen* or child* or p?ediat* or teenag* or young or youth*).ti.
7. "continuum of care"/ or aftercare/ or (aftercare or after care or continuity of care or follow up or transition*).ti.
8. 6 and 7
9. 4 or 5 or 8
10. best practices/ or professional standards/ or treatment guidelines/

11. (guideline* or clinical protocol* or clinical standard* or consensus statement* or framework* or model* or pathway* or policies or policy or practice* or "standard* of care" or statement*).ti,ab.
12. exp experimental design/
13. (trial* or study or rct).ti,ab.
14. 10 or 11 or 12 or 13
15. 3 and 9 and 14
16. (animal not human).po.
17. (book or dissertation abstract or edited book).pt.
18. 16 or 17
19. 15 not 18
20. limit 19 to (english language and yr="2020 -Current")

CINAHL (EBSCOhost)

S1. (MH "Neoplasms+") OR (MH "Oncology+") OR (MH "Cancer Patients") OR (MH "Cancer Survivors") OR (MH "Cancer Care Facilities")
S2. cancer* OR carcinoma* OR adenocarcinoma* OR blastom* OR glioblastom* OR glioma* OR leukaem* OR leukem* OR lymphom* OR malignan* OR melanoma* OR metastas* OR mesothelio* OR mesotelio* OR myeloma* OR neoplas* OR neuroblastom* OR oncolog* OR osteo#sarcom* OR sarcom* OR tumo#r* OR glioblastoma*
S3. S1 OR S2
S4. (MH "Transitional Care")
S5. TI transition* AND (TI adult* OR TI adolescen* OR TI child* OR TI p#ediat* OR TI teenag* OR TI young OR TI youth*)
S6. (transition* OR transfer*) N2 (adult* OR adolescen* OR child* OR p#ediat* OR teenag* OR young OR youth*)
S7. TI adolescen* OR TI child* OR TI p#ediat* OR TI teenag* OR TI young OR TI youth*
S8. ((MH "Child+") OR (MH "Adolescence") OR (MH "Young Adult")) AND (MH "Adult")
S9. S7 OR S8
S10. (MH "Continuity of Patient Care+")
S11. TI aftercare OR TI "after care" OR TI "continuity of care" OR TI "follow up" OR TI transition*
S12. S10 OR S11
S13. S9 AND S12
S14. S4 OR S5 OR S6 OR S13
S15. (MH "Practice Guidelines") OR (MH "Guideline Adherence")
S16. guideline* OR "clinical protocol*" OR "clinical standard*" OR "consensus statement*" OR framework* OR model* OR pathway* OR policies OR policy OR practice* OR "standard* of care" OR statement*
S17. (MH "Clinical Trials+") OR (MH "Prospective Studies") OR (MH "Nonexperimental Studies+")
S18. trial* OR study OR rct
S19. S16 OR S17 OR S18
S20. S3 AND S14 AND S19 [Publication Date: 20200101-; English Language]

Cochrane Library (Wiley)

#1. [mh Neoplasms] OR [mh "Medical Oncology"] OR [mh ^"Oncology Service, Hospital"] OR [mh ^"Oncology Nursing"] OR [mh ^"Cancer Survivors"]

#2. (cancer*:ti,ab OR carcinoma*:ti,ab OR adenocarcinoma*:ti,ab OR blastom*:ti,ab OR glioblastom*:ti,ab OR glioma*:ti,ab OR leukaem*:ti,ab OR leukem*:ti,ab OR lymphom*:ti,ab OR malignan*:ti,ab OR melanoma*:ti,ab OR metastas*:ti,ab OR mesothelio*:ti,ab OR mesotelio*:ti,ab OR myeloma*:ti,ab OR neoplas*:ti,ab OR neuroblastom*:ti,ab OR oncolog*:ti,ab OR osteo?sarcom*:ti,ab OR sarcom*:ti,ab OR tumo?r*:ti,ab OR glioblastoma*:ti,ab)

#3. #1 OR #2

#4. [mh ^"transition to adult care"]

#5. (transition*:ti AND (adult*:ti OR adolescen*:ti OR child*:ti OR p?ediat*:ti OR teenag*:ti OR young:ti OR youth*:ti))

#6. ((transition*:ti,ab OR transfer*:ti,ab) NEAR/2 (adult*:ti,ab OR adolescen*:ti,ab OR child*:ti,ab OR p?ediat*:ti,ab OR teenag*:ti,ab OR young:ti,ab OR youth*:ti,ab))

#7. (adolescen*:ti OR child*:ti OR p?ediat*:ti OR teenag*:ti OR young:ti OR youth*:ti)

#8. ([mh ^adolescent] OR [mh ^child] OR [mh ^"young adult"]) AND [mh ^adult]

#9. #7 OR #8

#10. [mh ^"continuity of patient care"] OR [mh ^aftercare] OR [mh ^"patient handoff"] OR [mh ^"patient transfer"] OR [mh ^"transitional care"] OR (aftercare:ti OR "after care":ti OR "continuity of care":ti OR "follow up":ti OR transition*:ti)

#11. #9 AND #10

#12. #4 OR #5 OR #6 OR #11

#13. [mh ^guideline] OR [mh ^"practice guideline"] OR [mh ^"Guideline Adherence"] OR [mh ^"Practice Guidelines as Topic"] OR [mh ^"Standard of Care"] OR [mh ^"Delivery of Health Care"] OR [mh ^"models, organizational"]

#14. (guideline*:ti,ab OR ("clinical" NEXT protocol*):ti,ab OR ("clinical" NEXT standard*):ti,ab OR ("consensus" NEXT statement*):ti,ab OR framework*:ti,ab OR model*:ti,ab OR pathway*:ti,ab OR policies:ti,ab OR policy:ti,ab OR practice*:ti,ab OR (standard* NEXT "of care"):ti,ab OR statement*:ti,ab)

#15. [mh ^"clinical study"] OR [mh "clinical trial"] OR [mh ^"clinical trial protocol"] OR [mh ^"observational study"] OR [mh ^"comparative study"] OR [mh ^"evaluation study"] OR [mh ^"multicenter study"]

#16. [mh ^"epidemiologic studies"] OR [mh "case-control studies"] OR [mh "cohort studies"]

#17. (trial*:ti,ab OR study:ti,ab OR rct:ti,ab)

#18. #13 OR #14 OR #15 OR #16 OR #17

#19. #3 AND #12 AND #18 [limited CENTRAL trial results to 2020-2025]

clinicaltrials.gov

Condition/disease: cancer

Other terms: transition to adult care

ANZCTR - Australian New Zealand Clinical Trials Registry
cancer AND transition to adult care

ICTRP - International Clinical Trials Registry Platform
cancer AND transition to adult care

ISRCTN - UK Clinical Study Registry
text search: cancer AND transition to adult care

Appendix 3: Full Search Strategies

Google Search

Prior to initiating the search, the browser cache and cookies were cleared to minimise personalised search bias and ensure retrieval of neutral, location-independent results. To maximise the number of retrievable results per page, the results per page was increased to 100 (maximum allowed via Google Search Settings). The URLs of the first 100 results were copied directly from the Google search results page. Using Microsoft Excel, the links were imported via, Data > Get Data > From Other Sources > From Web. This method generated a structured spreadsheet containing the page title; organisation; webpage URL; keywords; publication date; comments or metadata. The process was then repeated for the second set of 100 results, accessed by clicking "Next" at the bottom of the Google results page. The final dataset was saved, with 125 results provided using the search strategy. Two sheets were exported representing results 1–99 and 100–125 respectively. Rather than relying solely on metadata exported from the search results, each URL was accessed and its full content reviewed.

Targeted search

The following sites were used for the targeted search:

International and Regional Health Bodies:

- World Health Organization (WHO)
- Pan American Health Organisation (PAHO)

National and Specialist Cancer Organisations:

- Clinical Oncology Society of Australia (COSA)
- Cancer Council Australia
- Peter MacCallum Cancer Centre
- New South Wales Health
- Cancer Nurses Society of Australia
- Cooperative Trials Group for Neuro-Oncology (COGNO)
- Australian & New Zealand Children's Haematology/Oncology Group (ANZCHOG)

International Oncology and Supportive Care Networks:

- American Society of Clinical Oncology (ASCO)
- Children's Oncology Group (COG)
- International Society of Pediatric Oncology (SIOP)
- Multinational Association for Supportive Care in Cancer (MASCC)
- International Psycho-Oncology Society (IPOS)
- Association of Pediatric Oncology Social Workers (APOSW)

Charitable and Advocacy Organisations:

- Children with Cancer UK
- Childhood Cancer Association
- Children's Cancer Foundation
- World Child Cancer
- The National Children's Society
- St. Jude Children's Research Hospital

Sites were included if they were published within the previous five years (i.e., between April 2020 and April 2025). Freely accessible resources were reviewed with any guideline or resource behind paywalls or requiring membership access excluded; and guidelines were assessed for relevance based on title, scope, and inclusion of transition-related content.

Appendix 4: Data Extraction

Guidelines

General information was extracted, including the names of the authors, year of publication, journal or webpage source, country of origin, and the type of guideline. Additional data were collected regarding the target population, such as the types of cancer addressed, treatments and stages considered, age ranges, genders and sexes, inclusion of lived experience input, and the settings in which the guidelines were intended to be applied. Key recommendations and guidance were extracted across several domains, including preparation for transition, processes during transition, and care following transition. Further extraction focused on domains of care and the evidence base supporting each recommendation.

Trials and Trials Registries

General trial information was extracted, including the names of the authors, year of publication, contributing organisations, journal or webpage source, trial design and type, country in which the study was conducted, and details of the intervention such as its content, type, approach, schedule, and inclusion of lived experience input. Data were also extracted on the target population, including cancer types, number of participants, treatments and stages of cancer, age ranges, genders and sexes, and the settings in which the interventions were delivered. Outcomes were extracted across multiple dimensions, including efficacy, acceptability, feasibility, patient-reported outcome measures (PROMs), patient-reported experience measures (PREMs), adherence to follow-up care, qualitative feedback, and any other reported outcomes. As with the guidelines, key recommendations and guidance were extracted concerning preparation for transition, processes during transition, care following transition, domains of care, and the evidence supporting these recommendations.
